# Improving Tumor-Infiltrating Lymphocytes Score Prediction in Breast Cancer with Self-Supervised Learning

**DOI:** 10.3390/life14010090

**Published:** 2024-01-05

**Authors:** Sijin Kim, Kazi Rakib Hasan, Yu Ando, Seokhwan Ko, Donghyeon Lee, Nora Jee-Young Park, Junghwan Cho

**Affiliations:** 1 Department of Biomedical Science, Kyungpook National University, Daegu 41566, Republic of Korea; zmgpg123@knu.ac.kr (S.K.); krhasan02@knu.ac.kr (K.R.H.); yuando@knu.ac.kr (Y.A.); skanny@knu.ac.kr (S.K.); lowelkids24@knu.ac.kr (D.L.); 2 Department of Pathology, School of Medicine, Kyungpook National University, Daegu 41944, Republic of Korea; pathpjy@knu.ac.kr; 3 Department of Pathology, Kyungpook National University Chilgok Hospital, Daegu 41404, Republic of Korea; 4 Clinical Omics Institute, Kyungpook National University, Daegu 41405, Republic of Korea

**Keywords:** self-supervised learning, histopathology, breast cancer, tumor-infiltrating lymphocytes, tissue segmentation and lymphocyte detection

## Abstract

Tumor microenvironment (TME) plays a pivotal role in immuno-oncology, which investigates the intricate interactions between tumors and the human immune system. Specifically, tumor-infiltrating lymphocytes (TILs) are crucial biomarkers for evaluating the prognosis of breast cancer patients and have the potential to refine immunotherapy precision and accurately identify tumor cells in specific cancer types. In this study, we conducted tissue segmentation and lymphocyte detection tasks to predict TIL scores by employing self-supervised learning (SSL) model-based approaches capable of addressing limited labeling data issues. Our experiments showed a 1.9% improvement in tissue segmentation and a 2% improvement in lymphocyte detection over the ImageNet pre-training model. Using these SSL-based models, we achieved a TIL score of 0.718 with a 4.4% improvement. In particular, when trained with only 10% of the entire dataset, the SwAV pre-trained model exhibited a superior performance over other models. Our work highlights improved tissue segmentation and lymphocyte detection using the SSL model with less labeled data for TIL score prediction.

## 1. Introduction

Breast cancer is a prevalent tumor disease that frequently affects women before and after menopause, substantially disrupting their daily lives. Thus, multidisciplinary research is crucial to comprehend the risk factors associated with this form of tumor development [1]. Within this context, the tumor microenvironment (TME) plays a pivotal role in immuno-oncology, focusing on the intricate interplay between tumors and the human immune system. Notably, utilizing tumor-infiltrating lymphocytes (TILs) [2,3,4] as prognostic biomarkers in cancer patients can enhance immunotherapy precision, aiding in the removal of tumor cells in specific cancer types. Furthermore, breast cancer subtypes, such as Her2-positive and triple-negative breast cancer (TNBC), are focal subjects of extensive research into prognostic and predictive biomarkers.

This research aims to improve patient care and prognosis, as these subtypes are associated with the predominant challenges in breast cancer. According to Sheren Loi et al. [3,4], TILs are significant predictors in clinical TNBC studies. TIL identification and quantification can substantially refine treatment strategies, especially regarding immunotherapy precision, potentially reducing the need for more aggressive interventions like chemotherapy. To achieve accurate TIL detection, conducting comprehensive tissue segmentation and lymphocyte detection is imperative. This process includes identifying tumor regions and inflamed stroma associated with TILs [5].

Deep learning-based technologies have seen significant recent advancements within the medical field. These developments have positively impacted various medical domains, including radiology, histopathology, and genomics, by facilitating diagnoses and other critical tasks. Among these technologies, convolutional neural networks (CNNs) have emerged as the most widely adopted approach for medical image analysis [6,7]. However, despite their effectiveness, deep learning models require substantial amounts of labeled data for superior performance. Unfortunately, obtaining labeled data is time consuming and costly, posing practical challenges for training models on large datasets [8].

In response to these limitations, self-supervised learning methods [9,10,11,12,13] are garnering increasing attention within the research community. Self-supervised learning focuses on extracting meaningful features from input data through pretext tasks, introducing the distinct advantage of learning without requiring extensive labeled data. This shift towards self-supervised learning can potentially revolutionize how we approach deep learning-based medical applications, making them more accessible and cost effective, ultimately benefiting researchers and healthcare practitioners.

In this paper, we adopted a self-supervised learning approach by training our model using a substantial unlabeled pathology image dataset, eschewing ImageNet pre-trained weights. We leveraged the self-supervised pre-trained model to improve the performance of tissue segmentation and lymphocyte detection, finally leading to better TIL score prediction.

## 2. Related Works

Many research investigations have focused on classifying breast cancer images through various applications. Ayana et al. [14] introduced multistage transfer learning (MSTL), a breast cancer classification method. This approach utilizes three pre-trained models (EfficientNetB2, InceptionV3, and ResNet50) with three optimization algorithms (Adam, Adagrad, and stochastic gradient descent (SGD)) on deep learning techniques. In their study, the ResNet50-Adagrad setup achieved remarkable test accuracy rates of 99 ± 0.612% with the Mendeley dataset and 98.7 ± 1.1% with the MT-Small-Dataset. These findings were consistent across five cross-validation assessments, underlining the reliability of their approach.

Wang et al. [15] designed an innovative approach for automating cancer diagnosis and staging via image analysis and machine learning. Their study used the BreakHis dataset and employed preprocessing steps, including color-to-grayscale conversion, thresholding, and filtering. Nuclei segmentation was accomplished using distance transform and watershed algorithms, and they explored two feature extraction methods. The ensemble-tagged tree classifier achieved the highest binary classification accuracy at 89.7%, distinguishing between benign and malignant cases. Concerning multiclass classification, the ensemble subspace discriminant classifier achieved an 88.1% accuracy.

Similarly, Venugopal et al. [16] established a novel hybrid deep-learning approach employing Inspection-ResNetv2 and EfficientNetV2-S pre-trained models with ImageNet weights. Their model classified breast cancer histopathology images from the BreakHis and BACH datasets. The authors assessed their proposed model’s performance by comparing individual outcomes from Inspection-ResNetv2 and EfficientNetV2 models to the hybrid model’s output. The last classification layer contained four neurons for the BACH dataset and eight for the BreakHis dataset. Their results demonstrated the model’s effectiveness, achieving 98.15 percent precision with the BACH dataset and 99.03 percent with the BreakHis dataset.

Joshi et al. [17] introduced a deep CNN-based breast cancer detection method, evaluating three pre-trained CNN models (EfficientNetB0, ResNet50, and Xception) using the BreakHis and IDC datasets. Notably, the customized Xception model outperformed the others, achieving a 93.33% accuracy on 40× magnification images from the BreakHis dataset. The models were trained on 70% of the BreakHis dataset and validated on the remaining 30%, employing data augmentation, dropout, batch normalization, and other regularization techniques. Fine-tuning the improved Xception model on a subset of the IDC dataset yielded an 88.08% accuracy when detecting invasive ductal carcinoma. This study showcases the efficacy of transfer learning for diverse classification tasks on both datasets. Overall, the studies highlight the significance of image normalization and data augmentation and demonstrate transfer learning’s potential for increasing the accuracy of breast cancer classification systems.

Furthermore, we identified gaps in existing research regarding breast cancer for tissue segmentation, tumor detection, and TIL prediction. For instance, Amgad et al. [18] established a deep learning approach that simultaneously segments TILs at both region and nucleus levels. They employed fully convolutional neural networks (FCN-8) on top of the ImageNet pre-trained VGG16 architecture. The dataset used in this study comprised 120 anonymized H&E stained slides obtained from the Cleveland Clinic Foundation. However, their study had limitations, such as lacking validation on extensive image datasets and additional exploration of the relationship between spatial TIL features and biological data. Employing these techniques for comprehensive TIL analysis with whole slide images (WSIs) poses a challenge, as representative tumor regions on each slide must be manually selected.

To address limited training data, another study conducted by Lu et al. [19] developed a U-net based neural network to detect lymphocytic regions in H&E stained images. Inspired by its success in object detection, they utilized Resnet18 model’s initial five blocks as the encoder to enhance model robustness [20,21]. This choice was made to boost model efficiency and performance. Encoder parameters were initialized using pre-trained weights from ImageNet. This study utilized two breast cancer datasets: the TCGA-BRCA dataset and the lymphocyte detection dataset provided by Janowczyk et al. [22]. However, the trained network was still biased toward the training dataset, and the generalizability and transferability of the trained model remain in question. Leveraging self-supervised methods for transfer learning can address these challenges, offering the potential for automated and more comprehensive tissue segmentation and TIL analysis with whole slide images. Overall, these techniques ultimately advance the field of breast cancer diagnosis and treatment.

## 3. Materials and Methods

### 3.1. Background

During the TIGER challenge on the fully automated assessment of TILs in H&E stained breast cancer slides, we benchmarked tissue segmentation and lymphocyte detection performance with three teams among the top 10 ranked in leaderboards. Table 1 summarizes the methodology and results from teams (TiAger [23], Fda-Cdrh-Osel-Didsr, Xulin Chen) participating in the TIGER challenge. The goal of this challenge was two-fold: tissue segmentation and lymphocyte detection for computing an automated TIL score (leaderboard1) and to assess the prognostic value (C-index) of TIL scores (leaderboard2).

Additional information about the TIGER challenge and datasets can be found at https://tiger.grand-challenge.org/. The dataset was accessed on 16 January 2022.

Table 1 presents a common approach among research teams: the initialization of pre-trained weights from the ImageNet dataset [24,25] pre-trained models. ImageNet is an extensive dataset encompassing over 10 million natural images. Models pre-trained on this dataset have proven valuable in disciplines lacking specific training data. However, it is essential to note that ImageNet primarily comprises natural rather than pathological images, which are annotated. Fine-tuning models on natural domain image datasets, such as ImageNet, for subsequent pathological image analysis can alleviate some challenges posed by missing labeled data [26].

Rather than depending on only ImageNet pre-trained weights, ours is a groundbreaking approach in addressing limitations from a shortage of labeled data. We achieved more efficient learning by harnessing pre-trained weights derived from an extensive unlabeled dataset. This dataset included pathological images, particularly cancer tissue images from various organs, ensuring that the pre-trained weights were finely tuned to this specialized domain’s intricacies. We initialized our models using ImageNet pre-trained weights and trained the network with pathology datasets as the pre-training step.

After pre-training the encoders in a self-supervision-based model, we extracted and utilized weights from the pre-trained model’s encoders to initialize tissue segmentation and lymphocyte detection models. Next, we froze these encoders, preserving the knowledge acquired during pre-training. This technique ensures that the information gathered in the initial phase is retained and forms the foundation for subsequent analyses and tasks. We then froze the encoder weights and conducted a comparative analysis between models initialized with pathology image pre-trained weights, ImageNet pre-trained weights, and those initialized with random weights to assess their respective performances. Lastly, we selected the top-performing model for each downstream task and utilized it for TIL score prediction.

This approach allows us the leverage of domain-specific knowledge from unlabeled pathological images, offering a more tailored and efficient solution for TIL prediction.

### 3.2. Overall Pipeline for Til Score Prediction

**Figure 1:**
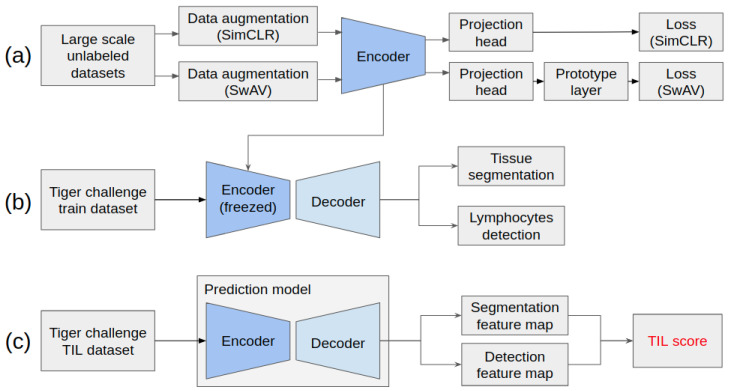
Overall pipeline for TIL prediction: (**a**) pre-training process using SimCLR and SwAV, (**b**) downstream analysis, and (**c**) TIL prediction.

In this work, we trained a self-supervised learning model using large-scale pathological datasets to obtain pre-trained encoder weights rather than relying on ImageNet pre-trained weights. We transferred these weights into the encoders of our downstream models for tissue segmentation and lymphocyte detection. The TIL score was calculated using the extracted tissue segmentation and lymphocyte detection information. Figure 1 illustrates the overall pipeline of this study.

Figure 1a details the SimCLR and SwAV training processes to pre-train encoders. In this step, we initialized the models using publicly available ImageNet weights. The weights of the pre-trained model encoders were transferred to the downstream encoders, and then the encoders were frozen. Figure 1b illustrates the learning process for downstream analysis tasks using the TIGER challenge datasets as inputs, encompassing tissue segmentation and lymphocyte detection. Figure 1c presents the feature maps generated by the models used as inputs for predicting the TIL score. These feature maps contain essential information extracted from the TIGER datasets.

### 3.3. Self-Supervision-Based Pretraining Task

During the initial phase of this study, we conducted pre-training by utilizing large-scale unlabeled pathological image datasets as input for self-supervised learning models. Specifically, we employed SimCLR and SwAV models.

#### 3.3.1. SimCLR

The Simple Framework for Contrastive Learning of Visual Representations (SimCLR) [27] is a self-supervised learning method designed to acquire meaningful representations using augmentation strategies as a pretext task. Every image undergoes augmentation with two distinct transformations in each mini-batch, including color or morphological changes, each with a specific probability. The encoder module is responsible for learning features from these augmented images. The encoder’s feature maps are then converted into embedding values through a projection head employing a multi-layer perceptron (MLP) neural network. These embedding values are then used with a contrastive loss function.

Concerning SimCLR, the augmented images from a single image are treated as positive pairs, while all other images are negative. The primary objective of SimCLR is to minimize the embedding distance between positive pairs while maximizing the distance between negative pairs, reducing overall loss. This contrastive learning technique proves highly effective when utilizing unlabeled data as input.

Nonetheless, there are certain limitations to consider. First, achieving optimal performance with SimCLR often necessitates a larger batch size, which can impose computational constraints and increase reliance on negative samples. Furthermore, self-supervised learning typically employs many datasets, often requiring multi-GPU training, which can introduce computational cost challenges [28].

#### 3.3.2. SwAV

Swapping Assignments between Views (SwAV) [29] is a self-supervised learning approach that leverages contrastive methods without computing pairwise comparisons. SwAV clusters data while ensuring that different augmentations of the same image share consistent cluster assignments. This process is accomplished through a novel “swapped prediction” mechanism, where the network predicts the cluster of one view using the representation of another. By doing so, SwAV eliminates the need for extensive memory banks [30,31], thus avoiding the computational cost of dissimilar data pair comparisons.

Notably, SwAV introduces a multi-crop augmentation strategy that further enhances model training efficacy. In traditional self-supervised learning, models typically augment one image into two, but SwAV incorporates additional multi-crop augmentations. In addition to the two standard augmented images, low-resolution cropped images undergo various augmentation techniques. While the comparison process evaluates the two standard augmented images against all augmented images, low-resolution cropped images are exclusively compared with standard augmented images.

This approach boosts performance by enabling comparisons between different views within a single image and addresses memory constraints, as low-resolution cropped images are smaller than the two standard augmented images. This strategy has the potential to enhance the performance of other techniques as well.

### 3.4. Downstream Analysis Task for Tissue Segmentation and Lymphocyte Detection

At the end of pre-training with a self-supervised learning model, the model weight is transferred and frozen into a downstream analytical model for tumor lymphocyte (TIL) prediction, followed by a segmentation and object detection learning process. We used DeepLabv3 for tissue segmentation and U-Net for lymphocyte detection.

#### 3.4.1. DeepLabv3 for Tissue Segmentation

In this study, DeepLabv3 [32] was applied among the DeepLab [33,34,35] series for tissue segmentation. It is a semantic segmentation technique that prominently features the Atrous Spatial Pyramid Pooling (ASPP) module as a core component.

The ASPP module uses multiple parallel filters by a dilated convolution with different stride rates. This dilated convolutional layer enlarges the receptive field by adjusting the stride within the filter of the extracted feature map. As a result, the ASPP module can capture multi-scale contextual features for each original image. These feature maps, extracted at various scales, are organized in parallel and are eventually fused into a single feature map, which is then produced as the output. Then, the output feature map undergoes bilinear upsampling to match the size of the original image. Utilizing the ASPP module of DeepLabv3 improves the performance of semantic segmentation.

#### 3.4.2. U-Net for Lymphocyte Detection

U-Net [36] is an encoder–decoder architecture widely used for image segmentation. In standard encoder–decoder models, encoders reduce input data dimensions, while decoders increase them, restoring high-dimensional images. However, this dimension encoder reduction can lead to the loss of detailed information from the original image. Even if decoders attempt to restore these lost data, they cannot fully recover them. Therefore, U-Net employs skip connections [37,38] that establish a direct link between the encoder and decoder layers to overcome this challenge. These connections facilitate data merging from the encoding and decoding stages, enabling the decoder to harness information from before and after dimension reduction phases. Consequently, U-Net excels at minimizing information loss during the encoding process, enhancing its ability to capture fine-grained details in image segmentation tasks.

### 3.5. Experiments

#### 3.5.1. Dataset and Data Preprocessing

Our study used three datasets for different tasks: (1) a dataset for pre-training self-supervised learning (SSL) models, (2) a WSIROIS dataset for tissue segmentation and lymphocyte detection, and (3) WSITILS for TIL prediction. The WSIROIS, WSITILS and their ground truths were derived from the TIGER challenge dataset. This dataset, including whole slide images (WSI), annotation files, and masks, was meticulously prepared and provided by the organizers of the TIGER challenge. The TIGER challenge itself was organized by the Diagnostic Image Analysis Group (DIAG) at Radboud University Medical Center in Nijmegen, Netherlands, in collaboration with the International Immuno-Oncology Biomarker Working Group. The annotations within this dataset were created under the guidance and expertise of qualified staff from these organizations, ensuring the accuracy and reliability of the ground truths. For detailed information regarding the dataset and the annotation process, additional insights can be found on the TIGER challenge website: https://tiger.grand-challenge.org/. The dataset was accessed on 16 January 2022. Additional details about the TIGER challenge datasets are summarized in Table S1 within Supplementary Materials.

We constructed a large-scale, unlabeled dataset to develop the pretrained model using self-supervised learning (SSL). This dataset was compiled from 15 publicly available sources. These sources include a diverse range of organ images, such as the breast, colon, bone, lung, and prostate, with further details provided in Table S2 of Supplementary Materials. Each of these datasets contains images of specific organ tissues, encompassing both normal and tumor regions. All images were uniformly cropped to 256 × 256 pixels for the inputs of the self-supervised model. The dataset comprised approximately 600,000 cropped images, which were randomly divided (80% for training and 20% for validation). The tissue segmentation task incorporated a WSIROIS dataset consisting of two distinct patch types images, namely ROI-level tissue Breast Cancer Semantic Segmentation (BCSS) and ROI-level tissue cells. The BCSS dataset consisted of 151 images, while the ROI-level tissue cell dataset comprised 1879 images, which were both employed for tissue segmentation. The dataset was originally annotated with masks covering eight classes, including invasive tumors, tumor-associated stroma, in situ tumors, healthy glands, non-in situ necrosis, inflamed stroma, rest, and background. However, these regions were merged into three broad categories for TIL prediction: tumor, stroma, and other.

For example, invasive tumors were grouped into the tumor class, while tumor-associated and inflamed stroma were merged into the stroma category. The remaining classes were collectively categorized as others. Similar to the preprocessing of the pretraining phase, we cropped these sub-WSI images to patch images with a uniform size of 256 × 256 pixels. This process resulted in approximately 19,000 cropped images that can be utilized as inputs for tissue segmentation. The dataset was partitioned, with 80% allocated for training and the remaining 20% for validation.

We utilized the ROI-level tissue cell dataset from the TIGER challenge for lymphocyte detection. This dataset includes annotated lymphocyte detection information stored in the ’tiger-coco.json’ annotation file, which details the bounding box coordinates of lymphocytes. To train our model for this task, we generated masks based on the provided annotation file, and these masks consisted of two classes: lymphocytes and background. Each bounding box within the masks was adjusted to 12 × 12 pixels, and both the input images and masks were cropped to uniform 256 × 256 pixels, following the same preprocessing procedure as the segmentation task. Images without lymphocytes were excluded from consideration, and smaller images were resized to 256 × 256 by adding zero-padding to the edges. Consequently, our dataset encompassed approximately 3195 images, with 80% allocated for training and 20% for validation.

#### 3.5.2. Experiment Setup of the Pre-Train Task and the Downstream Task

This paper employed ResNet-18 [38] as the backbone architecture for SimCLR and SwAV. ResNet [38] is a deep learning network with residual blocks enabling the model to tackle gradient vanishing/explosion challenges, allowing for the training of profoundly deep neural networks. Prior to the training process, we initialized encoders for SimCLR and SwAV with pre-trained weights from ImageNet, as this initialization scheme exhibited a better performance in training SSL models [24,25,39]. We separately trained the SimCLR and SwAV models for 300 epochs. During training, we selected the model with the lowest contrastive loss in the validation dataset. Each ResNet encoder from the best SSL model was frozen and used for downstream tasks. For these tasks, we utilized DeepLabv3 for tissue segmentation and U-Net for lymphocyte detection. We conducted 5-fold cross-validation for segmentation and detection. Furthermore, we conducted a series of experiments where we adjusted the learning rate within the range of [1.0 × 10^−3^, 1.0 × 10^−4^, 1.0 × 10^−5^] to determine the optimal learning rate for each downstream task. Subsequently, the downstream model was trained using the identified optimal learning rate. The experimental setup for pretraining and downstream tasks are detailed in Tables S3 and S4 in Supplementary Materials. The code for our TIL prediction pipeline is available at https://github.com/sijinkim2/TILs-prediction-pipeline. The website was accessed on 22 December 2023.

#### 3.5.3. Evaluation Metrics for Tissue Segmentation and Lymphocyte Detection

The Dice score [40] is the evaluation metric for tissue segmentation, measuring the degree of overlap between two images. For segmentation tasks, it assesses the performance of the model by quantifying the overlap between the ground truth and predicted images. The Dice score ranges from 0 (no overlap) to 1 (perfect overlap). In this study, we calculated individual Dice scores of tumor, stroma, and other classes.

In the lymphocyte detection task, we used the Free Response Operating Characteristic (FROC) curves [41] to assess performance. The FROC curve is the plot of sensitivity against the average number of false positives per image. The FROC score is calculated by the average sensitivity at six predefined false positives: [10, 20, 50, 100, 200, 300].

To predict TIL from the predictive mask, we filtered the mask by only retaining areas with a probability value exceeding 0.1. Next, a non-maximum suppression [42] technique based on distance was applied to ensure the selection of a single TIL within each bounding box. Considering that the bounding box size of the lymphocyte detection mask was 12, the distance threshold was also set to 12 units. Finally, the predicted detection results were compared with the measured results to calculate sensitivity and false positives. If the distance between the lymphocyte pixels of the predicted mask and the ground truth mask was within 8 pixels, it was considered true positive.

#### 3.5.4. Til Score Evaluation

For tumor-infiltrating lymphocyte (TIL) predictions, we employed the WSITILS dataset comprising 82 Whole Slide Image (WSI) type images. Then, each WSI image was resized to a uniform size of 256 × 256 pixels to facilitate TIL prediction. We excluded images with a background ratio exceeding 65%. The preprocessed images were fed into the best-performing models from tissue segmentation and lymphocyte detection tasks to serve as the TIL prediction model. We calculated the TIL scores using Equation (1), where TILs represented the number of lymphocytes within the stroma area. All datasets in the TIGER challenge comprised Whole Slide Images (WSIs) sampled at a resolution of 0.5 μ m/px. Furthermore, the average equivalent diameter of lymphocytes in these images was established as 8 μ m. Given this resolution and the size of lymphocytes, we calculated the lymphocyte size to be effectively represented in a 16 × 16 pixel area in our study.

(1) $$\mathrm{TIL}\;\mathrm{score}=100\times \frac{\sum (\mathrm{TILs}\times 16\times 16)}{\sum (\mathrm{stroma}\;\mathrm{area})}.$$

We evaluated performance using Pearson’s correlation coefficient between the predicted TIL score and the actual score provided in ‘tiger-til-score-wsitils.csv’. The ’tiger-til-score-wsitils.csv’ file contains the actual TIL score for 82 WSIs within the WSITILS dataset. Evaluation was conducted by a board-certified breast pathologist in adherence to the guidelines of the TIL Working Group. This csv file is included in the TIGER challenge training dataset. This dataset can be downloaded from https://tiger.grand-challenge.org/Data/. The dataset was accessed on 16 January 2022.

## 4. Results

### 4.1. Pre-Training of Simclr and Swav

In our experiment, we found that both self-supervised learning models exhibited a consistent trend where training loss decreased by epochs, as did validation loss. This observation indicates that overfitting did not occur during the learning process of these two self-supervised models. Detailed plots of the training and validation loss curves for the self-supervised learning models are available in Figure S2 in Supplementary Materials.

We selected the models with the lowest validation loss among the 300 epochs and extracted the encoder weights of ResNet18. The encoder weights were transferred to the DeepLabv3 encoder for tissue segmentation and to U-Net for lymphocyte detection.

### 4.2. Tissue Segmentation and Lymphocyte Detection

In this section, we present the results for tissue segmentation and lymphocyte detection, which are integral to predicting tumor-infiltrating lymphocyte (TIL) scores. Table 2 provides the overall results of these tasks and compares them with those of a randomly initialized model and an ImageNet pre-trained model. The ’randomly initialized model’ refers to a basic learning process that commences with weights initialized from scratch without pre-training. Conversely, the ’ImageNet pre-trained model’ incorporates weights initialized through pre-training on the ImageNet dataset. We obtained the publicly available ImageNet weights from TorchVision. The publicly available pre-trained weights were obtained and stored following the methods provided by He et al. [38]. Similar to the self-supervised pre-trained models in this paper, the weights of these models were frozen after initializing the encoders of the tissue segmentation and lymphocyte detection models. All results were averaged from a five-fold cross-validation.

Dice scores for the tumor and stroma class among the three classes during tissue segmentation are presented in Table 2. Notably, the random initialized model achieved the lowest Dice score. In contrast, the average Dice scores for the SimCLR and SwAV pre-trained models were 0.876 and 0.888, surpassing the ImageNet pre-trained model. The SwAV pre-trained model had the highest Dice score among all models.

**Figure 2:**
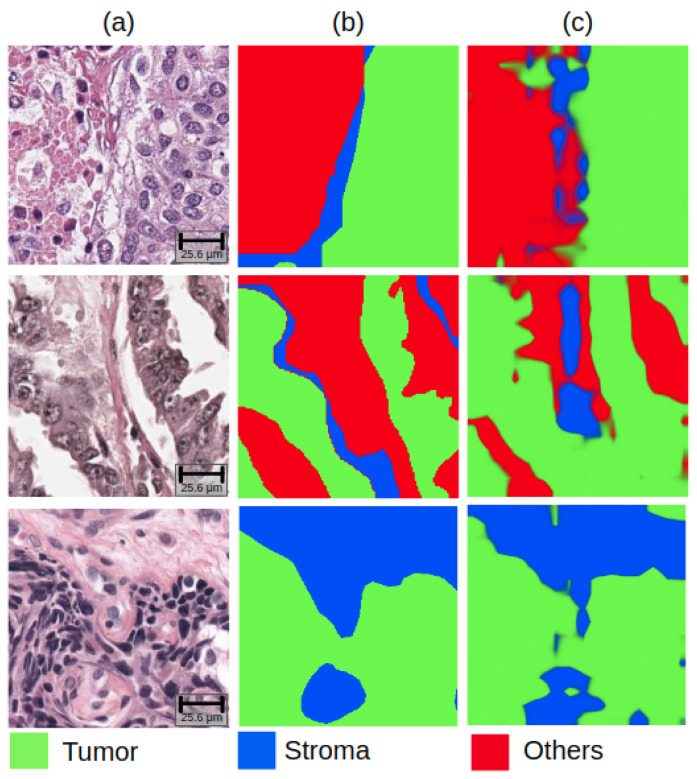
Representative examples of results on tissue segmentation task: (**a**) raw images, (**b**) ground truth masks, and (**c**) predictive tissue segmentation maps.

**Figure 3:**
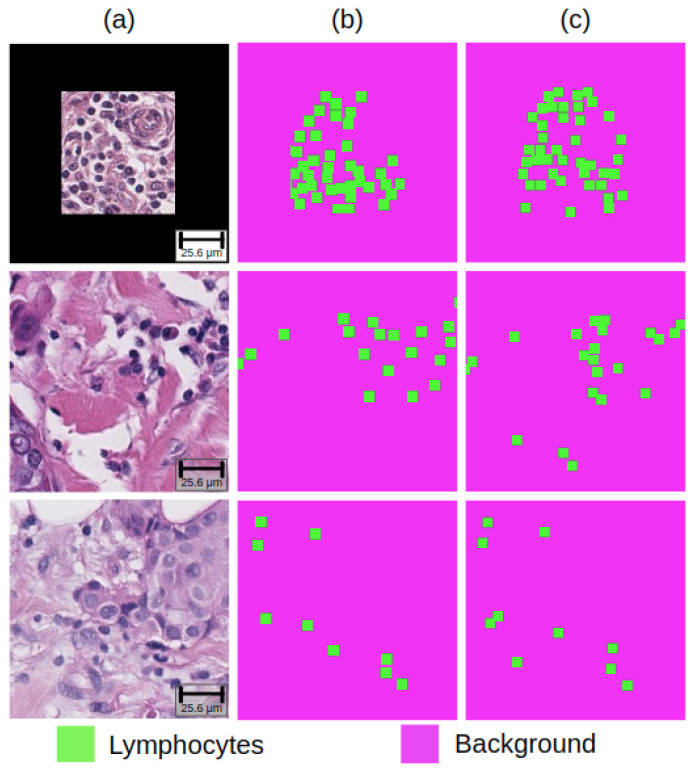
Representative examples of results on lymphocyte detection task: (**a**) raw images, (**b**) ground truth masks, and (**c**) predictive lymphocyte detection maps.

In the lymphocyte detection task, the randomly initialized model’s FROC score remained the lowest, while the SimCLR pre-trained model achieved the highest FROC score of 0.661 among models. Furthermore, the SimCLR pre-trained model’s FROC score was 2% higher than the ImageNet pre-trained model. Lastly, the SimCLR model marginally outperformed the SwAV model. Figure 2 and Figure 3 present the representative examples predicted by the best-performing model in each task.

### 4.3. Til Score Prediction

We utilized the best-performing models to predict tumor-infiltrating lymphocyte (TIL) scores: the SwAV pre-trained model for tissue segmentation and the SimCLR pre-trained model for lymphocyte detection (Table 2). Using the predicted lymphocyte map within the segmented stroma region in Equation (1), we calculated TIL scores for each slide. The predicted TIL scores against the actual scores yielded a Pearson correlation coefficient of 0.718, indicating a 4.4% higher value than that of the ImageNet pre-training model providing 0.674.

### 4.4. Performance on Size of Amount of Train Dataset

Table 2 indicates that self-supervised learning models achieved slightly higher performance than the ImageNet pre-trained model. These findings suggest that a substantial amount of training data positively influences all models, leading to comparable results.

To further investigate the influence of dataset size on the performance of the model, we conducted experiments by sampling the entire dataset with 10%, 50%, and 100% ratios. Experiments were conducted on the best folds for each model in Table 2, and the results can be found in Table 3. In the tissue segmentation task, the SwAV pre-trained model consistently outperformed the other models, achieving the highest Dice scores across all dataset sizes (10%, 50%, and 100%). The performance difference from the ImageNet pre-trained model was 1.9% when the dataset was at 100%, 2.4% at 50%, and 2.7% at 10%. For lymphocyte detection, the SimCLR pre-trained model achieved the highest FROC score when the dataset was set at 100% and 50%. However, when the dataset was reduced to 10%, the SwAV pre-trained model achieved the highest FROC score. Specifically, when the dataset was at 100%, the SimCLR pre-trained model’s FROC score peaked at 0.682, while the SwAV pre-trained model peaked at 0.54 when the dataset was 10%. Notably, the SwAV pre-trained model exhibited relatively good performance, outperforming other models even when the learning dataset was limited.

### 4.5. Performance on Fine-Tuned vs. Frozen Model

Next, we fine-tuned the model with the best performance for each task. The results of this fine-tuning process are presented in Table 4. In the tissue segmentation tasks, it was evident that all fine-tuned models outperformed the frozen models. We obtained the best performing model by fine-tuning the SwAV pre-trained weights, achieving Dice scores of 0.89 for tumors, 0.903 for stroma, and an average of 0.897. These results demonstrated that fine-tuning had a significant positive impact on tissue segmentation. However, for lymphocyte detection, the fine-tuned models, excluding the random initialized model, exhibited relatively lower FROC scores than the frozen models. Among the models presented in Table 4, the SimCLR pre-trained frozen model achieved the highest FROC score. This finding suggests that fine-tuning does not significantly improve lymphocyte detection, and frozen models perform better in this task.

## 5. Discussion and Conclusions

Instead of only using pre-trained weights from ImageNet, a large natural image dataset, we implemented self-supervised learning to improve tissue segmentation, lymphocyte detection, and tumor-infiltrating lymphocyte (TIL) prediction accuracy. We integrated self-supervised learning models (SimCLR and SwAV) with tissue segmentation and lymphocyte detection models (DeepLabv3 and U-Net). After evaluating the models, we consistently observed that the self-supervised pre-training models outperformed randomly initialized weight models and ImageNet pre-training models. The SwAV pre-training model achieved superior performance in tissue segmentation, while the SimCLR pre-training model demonstrated superior performance in lymphocyte detection. In particular, SwAV’s superior performance was noticeable with a limited dataset, emphasizing the methodology’s robustness and efficacy.

We used the best performing tissue segmentation and lymphocyte detection models to predict TIL scores. The predictive model achieved a 0.718 Pearson correlation coefficient, indicating a strong positive correlation with the actual TIL score. Consequently, the approach proposed in this paper improves the performance of two tasks for TIL prediction, as well as the performance of TIL score prediction. However, there are some limitations, and they are described as follows. First, it is important to note that while training the SimCLR model, our capacities were limited by a 1024 batch size due to memory constraint. To address this limitation in future studies, we plan to explore alternative self-supervised learning models less affected by batch size changes, such as BYOL (Bootstrap Your Own Latent) [43] and Dino [44]. Second, it is important to note that the number of cropped images in the pathology dataset discussed in this paper is approximately 600,000. This figure is not particularly large for a comprehensive vision foundation model. The inclusion of more diverse organs, beyond breast, colon, lung, and bone prostate, for learning purposes, could have further enhanced the results of tissue segmentation and lymphocyte detection. Such improvements would potentially make the findings applicable to downstream applications in other organ contexts beyond breast cancer. Third, all datasets in the TIGER challenge comprised whole slide images (WSI) that were downsampled to a resolution of 0.5 μ m/px. For lymphocyte detection tasks, we believe that using the original WSI before downscaling would yield more accurate detection results. In summary, this study underscores the efficacy of self-supervised learning, improving TIL score predictions and establishing a foundation for future advancements in medical imaging analysis. The outlined limitations provide avenues for further research and development in overcoming these challenges.

## Figures and Tables

**Table 1:** Summary of certain teams participating in the TIGER challenge.

	TiAger [23]	Fda-Cdrh-Osel-Didsr	Xulin Chen
Network	Efficient-U-Net	U-Net	HRNet-W18
Pre-trained model	ImageNet	ImageNet	ImageNet
Optimizer	Adam	Adam	SGD
Dice score (Tumor)	0.785	0.706	0.525
Dice score (Stroma)	0.790	0.772	0.563
FROC score	0.544	0.321	0.033
C-index	0.588	0.603	0.612

**Table 2:** Downstream results by a pre-trained model: DeepLabv3 for tissue segmentation and U-Net for lymphocyte detection. Bold values represent the best scores.

	Downstream Tasks			
	Dice Score of Tissue Segmentation			FROC Score of Lymphocyte Detection
	Tumor	Stroma	Average	
ImageNet pre-trained	0.861 ± 0.002	0.877 ± 0.003	0.869	0.641 ± 0.015
Random initialized	0.713 ± 0.005	0.746 ± 0.006	0.73	0.598 ± 0.015
SimCLR pre-trained	0.869 ± 0.003	0.883 ± 0.002	0.876	**0.661** ± 0.029
SwAV pre-trained	**0.882** ± 0.002	**0.894** ± 0.003	**0.888**	0.645 ± 0.013

**Table 3:** Downstream task results relative to dataset size. Bold values represent the best scores.

		Downstream Tasks			
		Dice Score of Tissue Segmentation			FROC Score of Lymphocyte Detection
	Fraction of Dataset	Tumor	Stroma	Average	
	10%	0.791	0.834	0.812	0.521
ImageNet pre-trained	50%	0.841	0.867	0.854	0.641
	100%	0.863	0.880	0.872	0.662
	10%	0.634	0.664	0.65	0.421
Random initialized	50%	0.704	0.721	0.713	0.531
	100%	0.72	0.751	0.736	0.617
	10%	0.817	0.841	0.829	0.523
SimCLR pre-trained	50%	0.846	0.869	0.858	0.653
	100%	0.872	0.886	0.879	**0.682**
	10%	0.831	0.848	0.839	0.54
SwAV pre-trained	50%	0.870	0.886	0.878	0.639
	100%	**0.884**	**0.898**	**0.891**	0.663

**Table 4:** Downstream task results comparing frozen and fine-tuned models. Bold values represent the best scores.

		Downstream Tasks			
		Dice Score of Tissue Segmentation			FROC Score of Lymphocyte Detection
		Tumor	Stroma	Average	
ImageNet	frozen	0.863	0.880	0.872	0.662
pre-trained	fine-tuned	0.870	0.890	0.880	0.645
Random	frozen	0.72	0.751	0.736	0.617
initialized	fine-tuned	0.812	0.843	0.828	0.623
SimCLR	frozen	0.872	0.886	0.879	**0.682**
pre-trained	fine-tuned	0.876	0.890	0.883	0.645
SwAV	frozen	0.884	0.898	0.891	0.663
pre-trained	fine-tuned	**0.890**	**0.903**	**0.897**	0.644

## Data Availability

The datasets used for the downstream task in this work are available from TIGER challenge (https://tiger.grand-challenge.org/). The dataset was accessed on 16 January 2022.

## Supplementary Materials

The following supporting information can be downloaded at: www.mdpi.com/xxx/s1, Table S1. Structure and content of training dataset of the TIGER challenge. Figure S1. Examples of rotated images and masks from the tissue-bcss dataset within WSIROIS: (a) raw image, (b) raw mask, (c) rotated and cropped image, (d) corresponding annotation mask. Table S2. Summary of large unlabeled datasets used for pre-training task. Table S3. Experiment setup of each self-supervised learning modules. Figure S2. SimCLR and SwAV loss curves obtained during pre-training. The red curve depicts SimCLR, and the blue depicts SwAV during (a) training and (b) validation. Table S4. Experiment setup of each downstream tasks. Figure S3. Validation loss curves for each downstream task relative to learning rate: (a) tissue segmentation and (b) lymphocyte detection. References [11,22,45,46,47,48,49,50,51,52,53,54,55] are cited in the Supplementary Materials.

## Abbreviations

TME	Tumor Microenvironment
TIL	Tumor-infiltrating Lymphocyte
SSL	Self-supervised Learning
TNBC	Triple-negative Breast Cancer
CNN	Convolutional Neural Network
FCN	Fully Convolutional Neural Network
MSTL	Multistage Transfer Learning
WSI	Whole Slide Image
TIGER	Tumor Infiltrating Lymphocytes in Breast Cancer
SimCLR	Simple Framework for Contrastive Learning of Visual Representations
MLP	Multi-layer Perceptron
SwAV	Swapping Assignments between View
ASPP	Atrous Spatial Pyramid Pooling
BCSS	Breast Cancer Semantic Segmentation
FROC	Free Response Operating Characteristic
BYOL	Bootstrap Your Own Latent
