# Supplementary material for: Improving Tumor-Infiltrating Lymphocytes Score Prediction in Breast Cancer with Self-Supervised Learning
Source: Life (Basel). 2024 Jan 5;14(1):0. doi: 10.3390/life14010090 (PMC11154396; doi:10.3390/life14010090)
Supplement: Supplementary file 1 [file life-14-00090-s001.zip › life-2732347-supplementary.pdf]

Supplementary Materials

Table S1 displays the training dataset used for tissue segmentation, lymphocyte detection, and TIL score prediction in this study. The training dataset of the TIGER challenge largely comprises WSIBULK, WSIROIS, and WSITILS. In this experiment, the WSIROIS dataset with ROI-level annotation (patch type) was used for tissue segmentation and lymphocyte detection.

Table S1. Structure and content of training dataset of the TIGER challenge.

Datasets	Data Configuration		Num_images	Use status
WSIBULK	images		92	not used
	tissue-masks		93	not used
	annotations-tumor-bulk	masks	93	not used
		xmls	93	not used
WSIROIS	WSI-level-annotation (WSI type)	images	196	not used
		tissue-masks	196	not used
		annotations-tissue-bcss-masks	151	not used
		annotations-tissue-bcss-xmls	151	not used
		annotations-tissue-cells-masks	168	not used
		annotations-tissue-cells-xmls	168	not used
	ROI-level-annotation (patch type)	tissue-bcss-images	151	used
		tissue-bcss-masks	151	used
		tissue-cells-images	1,879	used
		tissue-cells-masks	1,879	used
		tiger-coco.json	-	used
WSITILS	images		82	used
	tissue-masks		82	not used

	tiger-til-scores-wsitils.csv	-	used

Some tissue-bcss dataset within WSIROIS are annotated with only certain parts of the image as shown in (a) and (b) of Figure S1. Therefore, the image was rotated and cropped as shown in (c) and (d) of Figure S1 using the annotation information in the ‘annotation-tissue-bcss-xmls’.

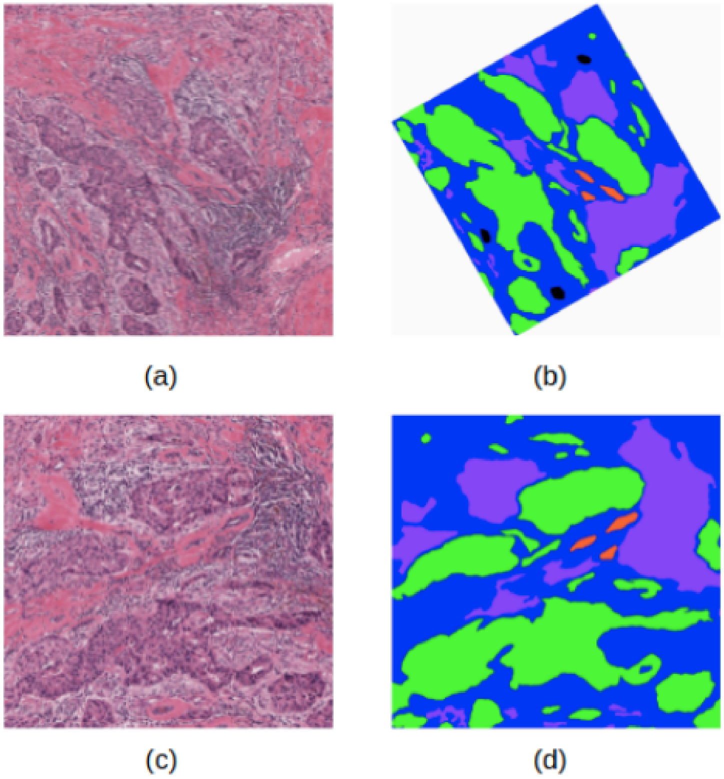

Figure S1. Examples of rotated images and masks from the tissue-bcss dataset within WSIROIS: (a) raw image, (b) raw mask, (c) rotated and cropped image, (d) corresponding annotation mask.

Table S2 provides basic information on the large unlabeled datasets used for training the pre-trained model in this work. These datasets consist of histopathological tissue images such as breast, colon, bone, lung, and prostate. All datasets are configured with a patch-type image, not a WSI-type.

Table S2. Summary of large unlabeled datasets used for pre-training task.

Dataset	Organ	Each image size	Number of images
andrewjanowczyk_epi[22]	Breast	1000 x 1000	125
andrewjanowczyk_mitosis[22]	Breast	2000 x 2000	311
andrewjanowczyk_nuclei[22]	Breast	2000 x 2000	142
andrewjanowczyk_tubule[22]	Colon	775 x 522	85
BACH[45]	Breast	2048 x 1536	500
breakhis[46]	Breast	700 x 460	9,109
breastpathq[47]	Breast	512 x 512	3,700
CoNSeP[48]	Colon	1000 x 1000	41
Gleason2019[49]	Prostate	5120 x 5120	331
Kather_texture_2016_image_tiles_5000[50]	Colon	150 x 150	5,000
LC25000[51]	Lung, Colon	768 x 768	25,000
Lymph[52]	Various	1388 x 1040	374
MIMM[53]	Bone	2560 x 1920	85
monuseg_2018_train_data[54]	Various	1000 x 1000	37
NCT-CRC-HE-100K[55]	Colon	224 x 224	100,000

Table S3 details the experimental setup for each self-supervised learning module used for pre-training. Regarding SimCLR, the maximum batch size was set to 1024 due to computation cost or GPU memory issues, and the SwAV batch size was set to 2048. The settings for augmentation methods were as follows: Random Horizontal Flip probability = 0.5, color jitter probability = 0.8, and Random Grayscale probability = 0.2. In our study, we adjusted the default color jitter values ([saturation, contrast, brightness, hue] =0.8, 0.8, 0.8, 0.4) into lighter ones([0.4, 0.4, 0.4, 0.2]). This modification was made based on insights from the study by Ozan Ciga et al.[11], particularly Appendix C and Figure C7. To multi-crop an image on SwAV, we used the default value (Number of crops: [2, 4], Min scale crop: [0.33, 0.10], Max scale crop: [1, 0.33]).

Table S3. Experiment setup of each self-supervised learning modules.

	SimCLR	SwAV
Backbone	ResNet-18	ResNet-18
Batch size	1024	2048
Epochs	300	300
Optimizer	Adam	Adam
Learning rate	1.0e-3	1.0e-3
LR scheduler	Cosine	Lambda
Weight decay	1.0e-6	1.0e-6
temperature	0.1	0.5
Epsilon(SwAV)	-	0.05
Sinkhorn iterations(SwAV)	-	3
Number of prototypes(SwAV)	-	28
Augmentation	- Color Jitter<br>- Random Grayscale<br>- Random Horizontal Flip<br>- Gaussian blur<br>- Random Resize crop	- Color Jitter<br>- Random Grayscale<br>- Random Horizontal Flip<br>- Gaussian blur<br>- Random Resize crop<br>- Multi crop

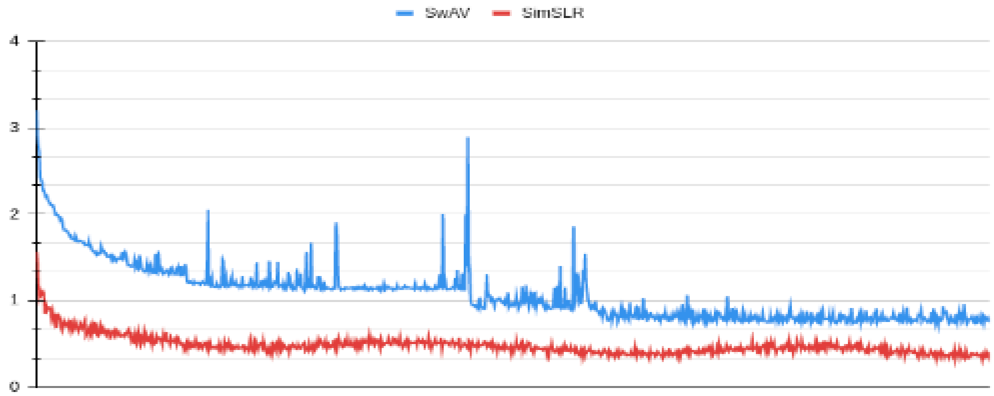

(a)

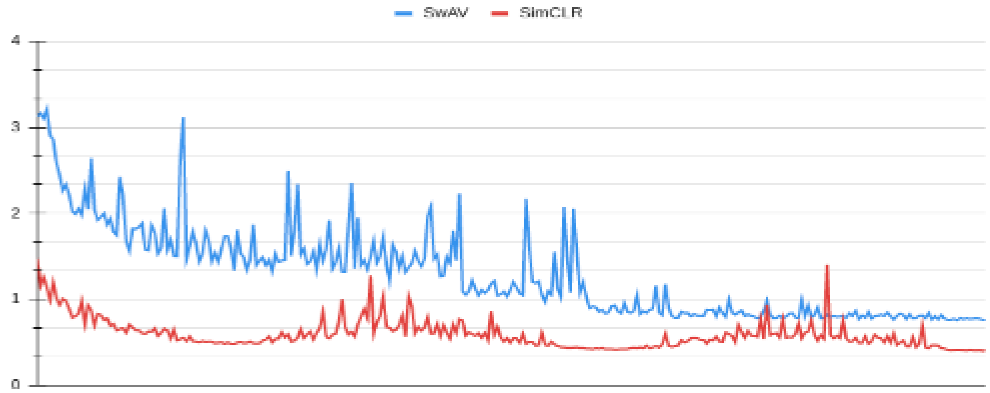

(b)

Figure S2. SimCLR and SwAV loss curves obtained during pre-training. The red curve depicts SimCLR, and the blue depicts SwAV during (a) training and (b) validation.

Table S4 details the experimental setup of tissue segmentation and lymphocyte detection tasks. While U-Net's encoder typically comprises general convolutional neural networks, we employed ResNet-18 as an encoder backbone. For tissue segmentation and lymphocyte detection, we performed experiments by sweeping the learning rate [1.0e-3, 1.0e-4, 1.0e-5] and selected the most optimal learning rate for each task. The optimal learning rate was 1.0e-4 for tissue segmentation and 1.0e-3 for lymphocyte detection. Figure S3 presents the validation loss curve according to the learning rate for each downstream task.

Table S4. Experiment setup of each downstream tasks.

	Tissue segmentation task	Lymphocytes detection task
Module	DeepLabv3	U-Net
Backbone	ResNet-18	ResNet-18
Batch size	40	15
Epochs	100	25
Optimizer	Adam	Adam
Learning rate	1.0e-4	1.0e-3
LR scheduler	Cosine	Cosine
Weight decay	1.0e-6	1.0e-6
Evaluation metric	Dice score	FROC score
Loss function	Dice BCE loss	BCE loss

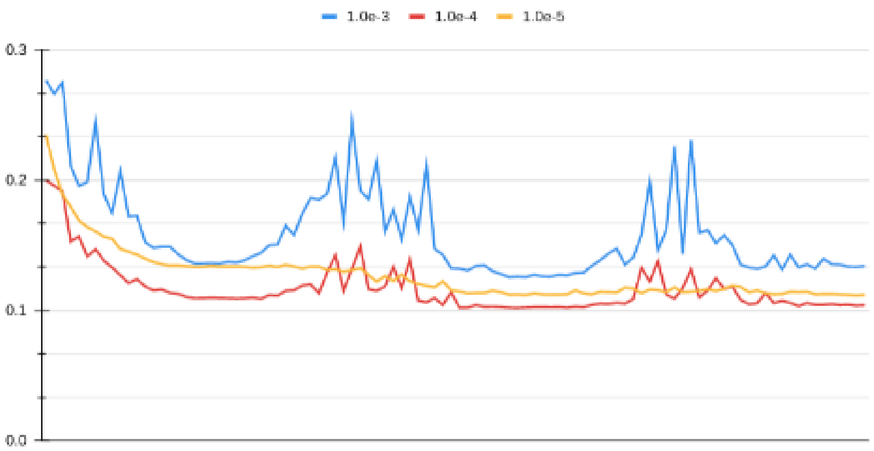

(a)

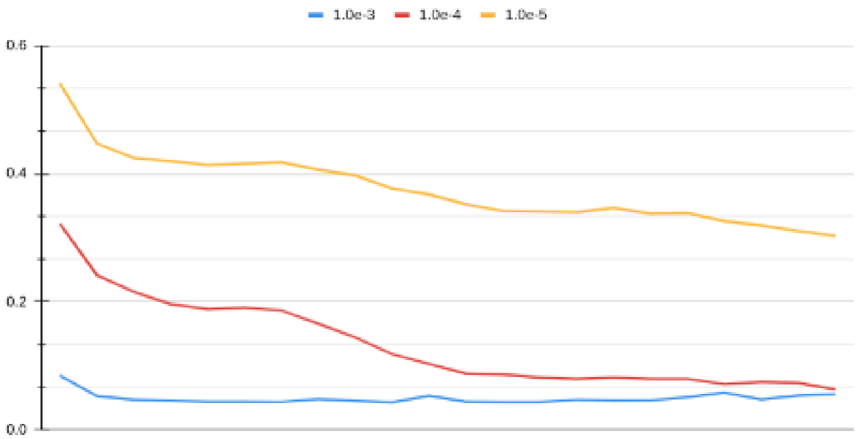

(b)

Figure S3. Validation loss curves for each downstream task relative to learning rate: (a) tissue segmentation and (b) lymphocyte detection.

Reference

11. Ciga, O.; Xu, T.; Martel, A.L; Self supervised contrastive learning for digital histopathology. *Machine Learning with Applications* **2022**, *7*, 100198
22. Janowczyk, A.; Madabhushi, A. Deep learning for digital pathology image analysis: A comprehensive tutorial with selected use cases. *Journal of pathology informatics* **2016**, *7*, 29
45. Aresta, G. et al. Bach: Grand challenge on breast cancer histology images. *Medical image analysis* **2019**, *56*, 122--139
46. Spanhol, F.A.; Oliveira, L.S.; Petitjean, C.; Heutte, L. A Dataset for Breast Cancer Histopathological Image Classification. *Ieee transactions on biomedical engineering* **2015**, *63*, 1455--1462
47. Akbar, S.; Peikari, M.; Salama, S.; Panah, A.Y.; Nofech-Mozes, S.; Martel, A.L. Automated and Manual Quantifcation of Tumour Cellularity in Digital Slides for Tumour Burden Assessment. *Scientific reports* **2019,** *9*, 14099
48. Graham, S.; Vu, Q.D.; Raza, S.E.A.; Azam, A.; Tsang, Y.W.; Kwak, J.T.; Rajpoot, N. Hover-net: Simultaneous segmentation and classification of nuclei in multi-tissue histology images. *Medical image analysis* **2019** , *58*, 101563
49. Nir, G. et al. Automatic grading of prostate cancer in digitized histopathology images: Learning from multiple experts. *Medical image analysis,* **2018***, 50,* 167--180
50. Kather, J.N. et al. Collection of textures in colorectal cancer histology. *Zenodo https://doi. org/10* **2016***, 5281*
51. Borkowski, A.A.; Bui, M.M.; Thomas, L.B.; Wilson, C.P.; DeLand, L.A.; Mastorides, S.M. Lung and Colon Cancer Histopathological Image Dataset (LC25000). *arXiv preprint arXiv:1912.12142* **2019**
52. Orlov, N.V.; Chen, W.W.; Eckley, D.M.; Macura, T.J.; Shamir, L.; Jaffe, E.S.; Goldberg, I.G. Automatic classification of lymphoma images with transform-based global features. *IEEE Transactions on Information Technology in Biomedicine* **2010**, *14*, 1003--1013
53. Gupta, R.; Gupta, A. MiMM_SBILab Dataset: Microscopic images of multiple myeloma. *The Cancer Imaging Archive* **2019**
54. Kumar, N. et al. A multi-organ nucleus segmentation challenge. *IEEE transactions on medical imaging* **2019**, *39*, 1380-1391
55. Kather, J.N; Halama, N.; Marx, A. 100,000 histological images of human colorectal cancer and healthy tissue. *Zenodo10* **2018**, *5281*
